# Impact of biologic therapy on blood cell indices and exacerbation risk in severe asthma: predictive value of the neutrophil-to-eosinophil ratio

**DOI:** 10.3389/falgy.2025.1691886

**Published:** 2025-11-07

**Authors:** Mona Al-Ahmad, Asmaa Ali, Wafaa Talat

**Affiliations:** 1 Department of Microbiology, College of Medicine, Kuwait University, Kuwait, Kuwait; 2 Department of Allergy, Al-Rashed Allergy Center, Ministry of Health, Kuwait, Kuwait; 3 Department of Laboratory Medicine, School of Medicine, Jiangsu University, Zhenjiang, China; 4 Department of Pulmonary Medicine, Abbassia Chest Hospital, Ministry of Health, Cairo, Egypt

**Keywords:** severe asthma, biologic therapy, neutrophil-to-eosinophil ratio, monocytes, exacerbation, biomarkers

## Abstract

**Background:**

Although biologic therapies have transformed the management of severe asthma, reliable blood-based markers to measure treatment response and predict residual exacerbation risk remain limited. The aim of this study was evaluating routine hematologic indices as predictors of disease control and exacerbations after biologic therapy.

**Methods:**

A cohort study included 107 patients with severe asthma were assessed before and after one year of initiating biologics. Asthma control was measured with the Asthma Control Test (ACT) and Asthma Control Questionnaire (ACQ-6); exacerbations were prospectively recorded. Complete blood counts were obtained at both time-points.

**Results:**

patients with severe asthma were predominantly middle-aged, obese, non-smoking women with poorly controlled asthma and elevated eosinophil counts. Biologic therapy resulted in a significant reduction in median blood eosinophil count, halving it from 480 to 240 cells/µl (*p* < 0.001). Smaller but statistically significant decreases were also observed in total leukocyte count and neutrophil count (both *p* = 0.02), leading to a marked increase in the neutrophil-to-eosinophil ratio (*N*ER, *p* < 0.001). Post-treatment, higher neutrophil counts and NER correlated with poorer asthma control, while elevated neutrophils, monocytes, and NER were significantly associated with exacerbations. Logistic regression confirmed monocytes (OR 1.03, *P* = 0.01) and NER (OR 1.07, *P* = 0.04) as independent predictors of exacerbation, with ROC analysis showing their significant discriminative ability (AUC 0.64-0.66). Depending on the clinical objective to rule out or confirm exacerbation risk, specific cutoffs for NER (>3.97) and monocytes (>435/µl) offered high sensitivity (∼92%), or high cutoffs for NER (>50.65) and monocytes (>755/µl) offered high specificity (∼91%).

**Conclusion:**

Biologic therapy significantly reduced eosinophils and altered NER in severe asthma. Prominently, elevated post-treatment neutrophils, monocytes, and particularly a higher NER, were significant predictors of poorer asthma control and increased exacerbation risk, offering clinically useful biomarkers for personalized management.

## Introduction

Asthma is a chronic airway disease characterized by inflammation, hyperresponsiveness, and variable airflow limitation. In genetically predisposed individuals, environmental triggers activate the airway epithelium, leading to the release of chemokines that attract inflammatory cells such as eosinophils, neutrophils, mast cells, and lymphocytes. This immune response causes tissue damage, mucus overproduction, and structural airway changes, forming the basis of asthma pathology (1–4).

Severe asthma affecting less than 10% of asthma patients and is a highly heterogenous disease that characterized by poor symptom control despite high-dose inhaled corticosteroids and additional controller medications, often requiring systemic corticosteroids (5, 6). It carries a disproportionate clinical and economic burden due to frequent exacerbations, hospitalizations, and reduced quality of life (7). Patients experience persistent symptoms, airflow limitation, and significant impairment in daily activities (6).

Severe asthma has two main inflammatory phenotypes: T2-high and T2-low. T2-high asthma, is the most recognized phenotype driven by type 2 helper (Th2) cells and innate lymphoid cells (ILC2), producing cytokines as IL-4, IL-5, and IL-13, These cytokines promote allergic inflammation and eosinophil recruitment. T2-high asthma is typically responsive to corticosteroids and targeted biologics such as anti-IgE and anti-IL-5 therapies. On the other hand, T2-low asthma involves neutrophilic or mixed granulocytic inflammation, often seen in older, obese, or smoking individuals. This phenotype is less responsive to standard therapies and has fewer targeted treatment options (8, 9).

Peripheral blood markers are valuable tools in asthma phenotyping and monitoring, offering a convenient, non-invasive means to assess underlying inflammation. Among these, blood eosinophil counts are well-established indicators of T2-high asthma and are routinely used to guide biologic therapy (10). Elevated eosinophil levels are associated with more severe disease and frequent exacerbations, and their reduction during treatment correlates with improved asthma control (11).

Biologic therapies, particularly those targeting IL-5 and IL-4/IL-13 pathways, effectively lower blood eosinophil counts (12). Patients with higher baseline eosinophil levels (typically >300 cells/μl) tend to experience more substantial reductions in exacerbation rates when treated with biologics (9, 12–14). Studies on mepolizumab have shown it not only depletes inflammatory eosinophil subsets but also reduces eosinophil granule proteins implicated in airway damage (15). While the magnitude of eosinophil reduction may vary among individuals, it remains a key marker of biologic response (10, 12).

In contrast, neutrophils commonly elevated in severe or T2-low asthma, are not directly targeted by current biologics (10, 16). Consequently, blood and sputum neutrophil levels often remain unchanged, particularly in patients with corticosteroid-resistant or non-eosinophilic inflammation (16). This is especially relevant in mixed or T2-low phenotypes, where neutrophil-driven inflammation plays a dominant role, and traditional therapies may be less effective (16, 17).

The neutrophil-to-eosinophil ratio (NER) has recently emerged as a promising composite biomarker, offering a broader perspective on the inflammatory milieu by reflecting the relative balance between neutrophils and eosinophils. Derived from routine blood counts, NER is simple to calculate and has been linked to poorer asthma control, reduced lung function, and increased exacerbation risk, particularly in patients with non-T2 or mixed inflammation (10).

Interestingly, biologic-induced eosinophil suppression may lead to a relative increase in NER, especially if neutrophil counts remain elevated (18–20). This shift might indicate a transition to a non-eosinophilic phenotype (20), though the clinical relevance of this observation is still being explored. Despite its potential prognostic utility, the role of NER in guiding treatment or monitoring response is not yet well defined, with no standardized thresholds or interpretation guidelines.

Generally, while eosinophil count remains the cornerstone for guiding biologic use, integrating neutrophil counts and NER could enhance our ability to identify patients with difficult-to-treat phenotypes, particularly those with T2-low inflammation (19, 20). However, comprehensive data on the impact of biologics on neutrophils and NER, especially in non-T2 asthma, remain limited and warrant further investigation. From that point of view, this study designed to evaluate the role of routine hematologic indices, particularly, blood neutrophils and NER as a predictive biomarkers of disease control and exacerbations risks following biologic therapy.

## Methods

### Patients, study design and sample size

A cohort study was conducted including adult patients (≥18 years) with a confirmed diagnosis of severe asthma, as defined by the European Respiratory Society (ERS)/American Thoracic Society (ATS) guidelines (5). Baseline demographic, clinical, and laboratory data were collected retrospectively from medical records, while exacerbations and treatment outcomes were prospectively documented over a one-year follow-up. All patients were biologic-naïve at baseline and eligible to initiate add-on biologic therapy at our center. To ensure adequate exposure, only patients who received at least 12 months of continuous biologic treatment were included, with adherence confirmed by administration of all injections at the center (no home prescriptions).

Patients received biologic therapy tailored to their clinical phenotype and biomarker profile, including anti-IgE (omalizumab), anti-IL-5/IL-5R (mepolizumab, benralizumab), or anti-IL-4R*α* (dupilumab). Omalizumab was prescribed for patients with elevated IgE and allergic asthma; mepolizumab or benralizumab for those with elevated blood eosinophils (≥150 cells/μl) and frequent exacerbations; and dupilumab for patients with CRSwNP, atopic dermatitis, or persistent type 2 inflammation despite other therapies. No T2-low patients were included.

Exclusion criteria included active respiratory infection within 4 weeks prior to enrollment, systemic corticosteroid use for non-asthma indications, comorbid bronchiectasis, and malignancy.

The sample size was calculated using Minitab version 17.1.0.0, based on an estimated prevalence of severe asthma of less than 1% in Kuwait (given that the overall asthma prevalence is 9.6%, with fewer than 10% of cases classified as severe) (21–24). The calculation ensured 80% statistical power and a 90% confidence level, while controlling for both Type I (*α* = 0.05) and Type II (*β* = 0.2) errors. The minimum required sample size was estimated to be approximately 93 patients.

### Ethics approval and consent to participate

The study received approval from the Kuwait Ministry of Health Ethical Committee (Approval Number 2256/2023), aligning with local guidelines and the Helsinki Declaration. All participants provided written informed consent, ensuring their voluntary participation and understanding of the study, thus upholding ethical conduct and global research standards.

### Data collection and study end point

Data were collected from medical records and included demographic characteristics such as age, sex, smoking status, and comorbidities. Clinical and functional data comprised the Asthma Control Test (ACT), and the Asthma Control Questionnaire (ACQ-6). Additional parameters included the number of asthma exacerbations and oral corticosteroid (OCS) courses per year, spirometry results (post-bronchodilator FEV1% predicted, FVC% predicted, and FEV1/FVC%), and differential blood cell counts with calculation of the neutrophil-to-eosinophil ratio (NER).

All data were collected at two time points: baseline (immediately before starting biologic therapy) and follow-up (after one year of treatment). Comparisons between baseline and one-year follow-up data were used to assess the impact of biologic therapy on blood cell indices. Disease exacerbation was defined according to standard criteria as a significant worsening of asthma symptoms and lung function requiring systemic corticosteroids for at least three days, an emergency department visit, or hospitalization (1, 25). Exacerbations were prospectively recorded through clinician verification during routine follow-up visits and confirmed via medical records. Post-treatment blood samples were collected at least four weeks after any systemic corticosteroid course, and patients were required to be clinically stable at the time of sampling to avoid acute treatment effects.

### Statistical analysis

Data were entered into an Excel spreadsheet and analyzed using Minitab version 17.1.0.0 for Windows (Minitab Inc., 2013, Pennsylvania, USA). The Shapiro–Wilk test was used to assess the normality of the data. To compare median values before and after biologic therapy, Wilcoxon signed-rank test was applied. Logistic regression analysis with a forward selection method was conducted to identify independent predictors of asthma exacerbation. The discriminative performance of blood biomarkers for predicting exacerbation was evaluated using receiver operating characteristic (ROC) curve analysis, with an area under the curve (AUC) greater than 0.6 considered acceptable. All statistical tests were two-sided, with a significance level set at ≤0.05.

## Results

Table 1 presented the baseline characteristics of patients with severe asthma prior to initiating biologic therapy. The cohort primarily consisted of middle-aged to older adult females, most of whom were non-smokers but frequently obese. A notable allergic profile was observed, marked by high rates of allergic rhinitis and nasal polyps. Nearly 75% of patients had adult-onset asthma. Baseline ACT and ACQ-6 scores indicated poor asthma control, while lung function tests confirmed moderate to severe airflow limitation. Elevated eosinophil counts in many patients supported eligibility for eosinophil-targeted therapies. Additionally, the wide, right-skewed distribution of the Neutrophil-to-Eosinophil Ratio (NER) highlights its potential as a predictive biomarker.

**Table 1:** Demographic and clinical features of patients with severe asthma before biologic therapy.

Factors	Total (*n* = 107)				
	Mean	SD	Median	Q1	Q3
Age	54.55	11.52	55	47	64
BMI	31.26	5.30	31	28	34
Sex	N	%			
Female	74	69.16			
Male	33	30.84			
Smoking	N	%			
Ex-smoker	10	9.35			
Non-smoker	91	85.05			
Smoker	6	5.61			
Disease onset	N	%			
Adult-onset	79	73.83			
Childhood-onset	28	26.17			
Comorbidity					
AR	N	%			
No	13	12.15			
Yes	94	87.85			
NP	N	%			
No	47	43.93			
Yes	60	56.07			
Eczema	N	%			
No	99	92.52			
Yes	8	7.48			
DM	N	%			
No	87	81.31			
Yes	20	18.69			
HTN	N	%			
No	91	85.05			
Yes	16	14.95			
Hypothyroid	N	%			
No	96	89.72			
Yes	11	10.28			
Patient's parameters	Mean	SD	Median	Q1	Q3
ACT	12.78	4.86	12	8	17
ACQ-6	2.01	1.27	1.80	0.80	3
FEV1	57.35	19.52	55.1	44.8	67.9
FVC	63.66	16.63	60.8	53.9	73.3
FEV1: FVC	75.517	9.339	77.2	68.3	83.1
Blood cells and indices	Mean	SD	Median	Q1	Q3
TLC	8,026	2,405	7,640	6,210	9,840
Neutrophil	4,370	1,838	4,160	3,170	5,006
Lymphocyte	2,469.2	825.7	2,410	1,860	2,840
BEC	568.9	422.8	480	260	790
Monocyte	595.4	244.6	550	440	680
PLT	315.78	80.3	313	257	365
Hb	131.97	17.27	133	123	144
NER	22.63	101.72	8.69	5.07	15.31

The numerical data presented as mean and standard deviation, median and inter quartile range (Q1-Q3), and categorical data as number and percentage.

N, number; SD, standard deviation; Q1, quartile 1; Q3, quartile 3; BMI, body mass index; AR, allergic rhinitis; NP, nasal polyp; DM, diabetes mellitus; HTN, hypertension; ACT, asthma control test; ACQ, asthma quality of life questionnaire; TLC, total leukocytic count; BEC, blood eosinophil count; PLT, platelets; Hb, hemoglobin; NER, neutrophil: eosinophil ratio.

**Figure 1:**
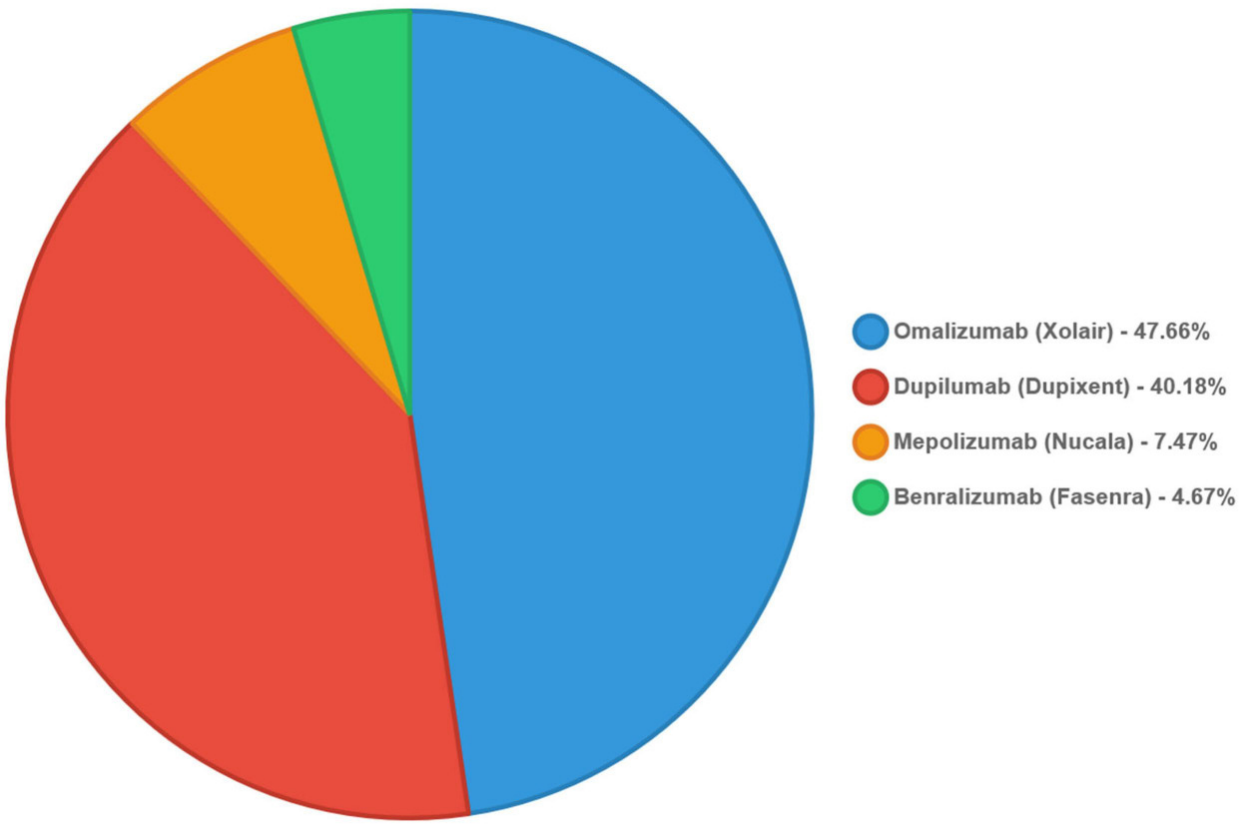
Distribution of prescribed biologics.

Figure 1 illustrated the distribution of biologic therapies among the patient cohort. The data showed that the predominant use of omalizumab in this population, followed by dupilumab, with lower utilization of mepolizumab and benralizumab. Hence, the most frequently prescribed biologic was omalizumab (Xolair), used in 51 patients (47.66% of the total). Dupilumab (Dupixent) was the second most common, prescribed to 43 patients (40.18%). Mepolizumab (Nucala) and benralizumab (Fasenra) were less frequently used, with 8 (7.47%) and 5 (4.67%) patients, respectively.

Table 2 illustrated the impact of biologic therapy on different blood cells and indices, a statistically significant reduction was noted in the TLC and neutrophil count (both *p* = 0.02), suggesting a suppressive effect on overall leukocytic and neutrophilic inflammation. The BEC showed the most pronounced decline (median reduced from 480 to 240, *p* < 0.001), a finding consistent with the mechanism of action of biologics that target type 2 inflammation. Interestingly, the neutrophil-to-eosinophil ratio (NER) significantly increased after treatment (*p* < 0.001), which reflects the disproportionately greater reduction in eosinophils compared to neutrophils. Other hematological parameters, including lymphocyte count, monocyte count, PLT, and Hb, showed no statistically significant changes, indicating that the biologic therapy's effect is selective toward eosinophils and neutrophils rather than causing broad hematological alterations. Overall, biologic therapy effectively reduces eosinophilic inflammation, with parallel but smaller decreases in TLC and neutrophils. The marked rise in NER is a key finding, underscoring eosinophil suppression as the dominant hematologic signature of this treatment. Clinically, this selective dampening of type 2 inflammation aligns well with the therapeutic goal of biologics in severe asthma and related diseases, and it's achieved without inducing generalized cytopenia.

**Table 2:** Impact of biologic therapy on blood indices.

Factors	Total before (*n* = 107)					Total after (*n* = 107)					*P* ^a^
Blood cells and indices	Mean	SD	Median	Q1	Q3	Mean	SD	Median	Q1	Q3	
TLC	8,026	2,405	7,640	6,210	9,840	7,524	2,242	7,230	5,920	9,110	**0.02**
Neutrophil	4,370	1,838	4,160	3,170	5,006	4,034	1,716	3,750	2,760	5,040	**0.02**
Lymphocyte	2,469.2	825.7	2,410	1,860	2,840	2,458	905	2,270	1,870	2,940	0.71
BEC	568.9	422.8	480	260	790	542	1,038	240	120	660	**<0.001**
Monocyte	595.4	244.6	550	440	680	563.8	181.4	530	450	640	0.29
PLT	315.78	80.3	313	257	365	314.88	77.57	304	264	360	0.53
Hb	131.97	17.27	133	123	144	131.07	16.62	132	123	142	0.49
NER	22.63	101.72	8.69	5.07	15.31	33	107.1	12.5	5	27.8	**<0.001**

The numerical data presented as mean and standard deviation, median and inter quartile range (Q1-Q3). The bold number denotes statistically significant value.

N, number; SD, standard deviation; Q1, quartile 1; Q3, quartile 3; TLC, total leukocytic count; BEC, blood eosinophil count; PLT, platelets; Hb, hemoglobin; NER, neutrophil: eosinophil ratio.

a The test of significant: Wilcoxon signed-rank test, *p* < 0.05 considered significant.

**Figure 2:**
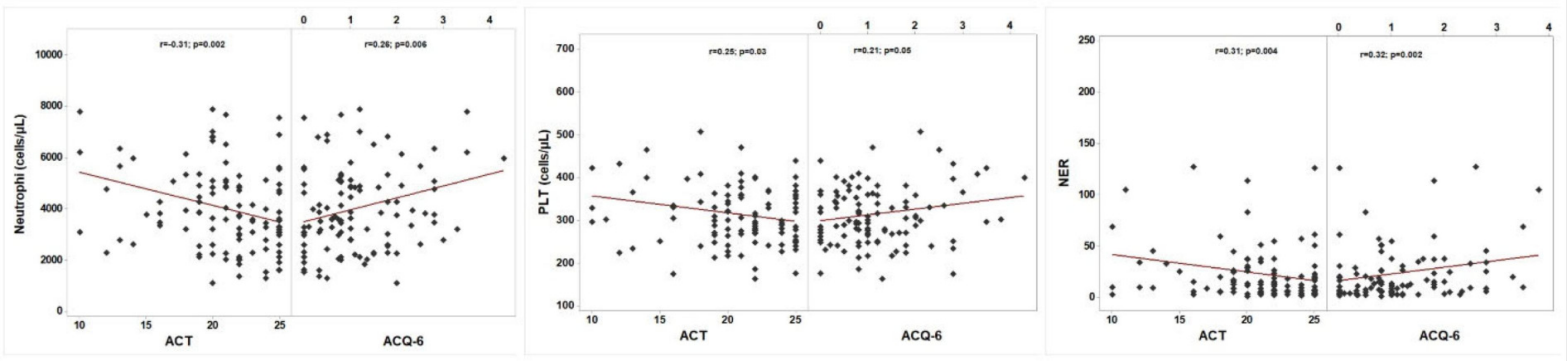
Blood indices in correlation with asthma control parameters after biologic therapy.

Moreover, following biologic therapy, blood cell indices exhibited varying degrees of correlation with asthma control. Remarkably, higher neutrophil counts and an elevated NER showed significant association with poorer asthma control, as reflected by lower ACT scores and higher ACQ-6 scores (Figure 2). Additionally, both total TLC and PLT demonstrated significant negative correlation with ACT scores. Interestingly, although biologic therapy led to a marked reduction in eosinophil counts, the post-treatment blood eosinophil count (BEC) did not significantly correlate with asthma control measures.

Table 3 revealed that, among patients with severe asthma following biologic therapy, certain blood indices were significantly associated with asthma exacerbations. Patients who experienced exacerbations had significantly higher neutrophil counts (*P* = 0.02) and markedly elevated monocyte counts (*P* = 0.007) compared to those with controlled asthma. Importantly, the Neutrophil-to-Eosinophil Ratio (NER) was also significantly higher in the exacerbation group (*P* = 0.04). In contrast, there were no statistically significant differences between the two groups in total leukocyte count, lymphocyte count, blood eosinophil count (BEC), platelet count, or hemoglobin levels.

**Table 3:** Blood indices in correlation with asthma exacerbation.

Factors	Controlled Asthma (*n* = 69)					Asthma Exacerbation (*n* = 38)					*p*
Blood cells and indices	Mean	SD	Median	Q1	Q3	Mean	SD	Median	Q1	Q3	
TLC	7,266	2,102	6,960	5,495	8,805	7,993	2,435	7,840	6,200	9,618	0.16
Neutrophil	3,755	1,526	3,450	2,575	4,850	4,540	1,937	4,355	3,198	5,735	**0.02** ^a^
Lymphocyte	2,455.3	713.7	2,270	1,905	2,930	2,463	1,188	2,295	1,680	3,001	0.54
BEC	532.2	578.4	280	125	765	559	1,572	215	90	430	0.17
Monocyte	531.9	153.7	510	430	600	621.9	213.2	580	520	660	**0.007** ^a^
PLT	306.59	64.34	299	262.5	358.5	329.9	96.3	314.5	268.5	384.8	0.35
Hb	131.16	17.39	133	123.5	141	130.92	15.35	130	119.75	145	0.67
NER	19.53	24.79	10.76	3.84	25.69	31.02	43.53	16.65	8.3	36.5	**0.04** ^a^

The numerical data presented as mean and standard deviation, median and inter quartile range (Q1-Q3). The bold number denotes statistically significant value.

N, number; SD, standard deviation; Q1, quartile 1; Q3, quartile 3; TLC, total leukocytic count; BEC, blood eosinophil count; PLT, platelets; Hb, hemoglobin; NER, neutrophil: eosinophil ratio.

a The test of significant: Mann Whitney test, *p* < 0.05 considered significant.

Table 4, derived from logistic regression analysis, identified monocyte count and the NER as statistically significant predictors of asthma exacerbation. Each unit increase in monocyte count was associated with a 3% increase in the odds of exacerbation (OR=1.03, *P* = 0.01), while each unit increase in NER corresponded to a 7% increase in the odds (OR = 1.07, *P* = 0.04). Other variables, including platelet count, age, sex, allergic rhinitis, and nasal polyps, did not emerge as significant predictors in the model.

**Table 4:** Predictors of asthma exacerbation.

Factors	CE	OR	95% CI	*P* ^a^
Monocyte	0.03	1.03	(1.0006, 1.0061)	**0.01**
PLT	0.04	1.04	(0.9984, 1.0097)	0.15
NER	0.07	1.07	(0.9939, 1.0217)	**0.04**
Age	−0.01	1.00	(0.9572, 1.0424)	0.96
Sex (M)	−0.18	0.84	(0.2769, 2.5409)	0.76
AR	0.45	1.57	(0.3474, 7.0633)	0.55
NP	0.31	1.36	(0.4625, 3.9969)	0.57

The bold number denotes statistically significant value.

PLT, platelets; NER, neutrophil: eosinophil ratio; M, male; AR, allergic rhinitis; NP, nasal polyps; CE, coefficient; OR, odd ratio; CI, confidence interval, the test of fitness: Hosmer Lemeshow-Test, X^2^ = 12.5, *p* = 0.13.

^a^The test of significant: Logistic regression with stepwise forward selection methods, *p* < 0.05 considered significant.

**Figure 3:**
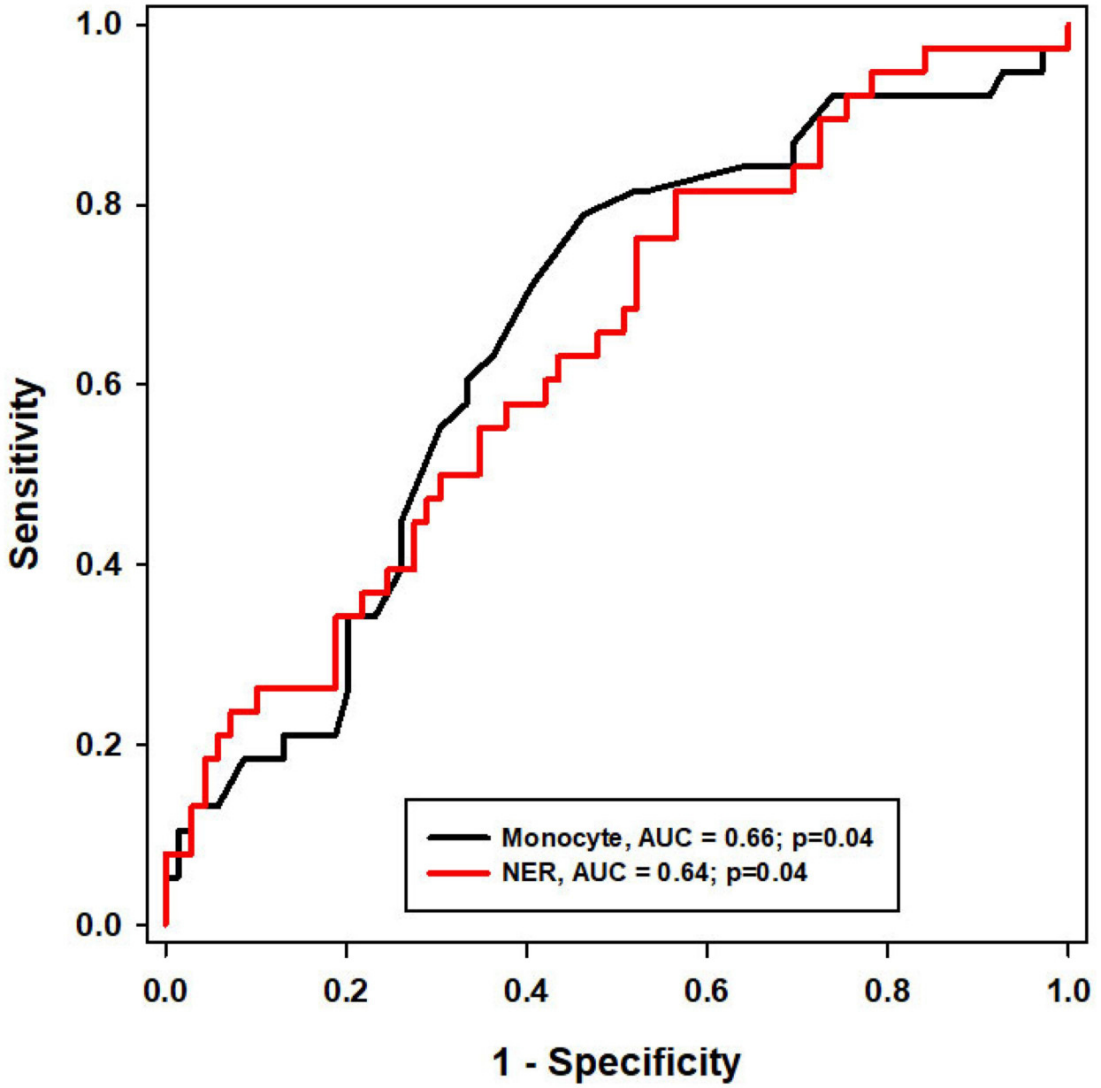
ROC curve of monocyte and NER for discrimination of asthma exacerbation.

Figure 3 displayed ROC curves for monocyte count and the NER and showed that both biomarkers had modest but statistically significant discriminatory power in predicting asthma exacerbations following biologic therapy (AUC = 0.66 and 0.64, respectively; both *P* = 0.04). Table 5 further supported their predictive utility by presenting performance metrics across various cutoff values. Lower cutoffs for monocyte count (>435) and NER (>3.97) provided high sensitivity (92%), making them suitable for screening or ruling out exacerbations, with negative predictive values (NPV) around 76%–77%. However, this came at the expense of low specificity and only moderate positive predictive value (PPV ∼55%). In contrast, higher cutoffs for monocyte count (>755) and NER (>50.65) achieved high specificity (91%) and stronger PPVs (68% for monocyte, 73% for NER), making them more effective for confirming exacerbation risk, despite their poor sensitivity. This analysis suggested that the choice of cutoff value depended on whether the clinical objective was to rule out or confirm the likelihood of exacerbation.

**Table 5:** Utility of monocyte and NER in predicting asthma exacerbation.

Cutoff >		Sensitivity	95% CI	Specificity	95% CI	PPV	NPV
Monocyte	435	92%	0.7862 to 0.9834	26%	0.1625 to 0.3806	55%	77%
	755	18%	0.07743 to 0.3433	91%	0.8203 to 0.9674	68%	53%
NER	3.97	92%	0.7862 to 0.9834	25%	0.1505 to 0.3649	55%	76%
	50.65	24%	0.1144 to 0.4024	91%	0.8203 to 0.9674	73%	54%

NER, neutrophil: eosinophil ratio; CI, confidence interval; PPV, positive predictive value; NPV, negative predictive value.

## Discussion

The neutrophil-to-eosinophil ratio (NER), along with other indices such as the neutrophil-to-lymphocyte ratio (NLR), has emerged as a promising biomarker in asthma. Elevated NER and NLR values have been associated with poor disease control, increased exacerbation risk, higher hospitalization rates, and reduced lung function (26–28). These indices offer practical tools for clinical risk stratification, particularly in identifying patients with uncontrolled or severe asthma.

In parallel, biologic therapies have significantly transformed severe asthma management by selectively targeting eosinophilic inflammation. Agents such as mepolizumab, benralizumab, and dupilumab reduce blood eosinophil counts, improve symptom control, reduce exacerbations, and enhance quality of life (29, 30). These therapies act via mechanisms including IL-5 inhibition and blockade of eosinophil recruitment, while sparing other immune cells, especially neutrophils (18). As a result, although the absolute neutrophil count remains stable, the NER typically increases following biologic initiation, thus reflecting eosinophil depletion rather than neutrophil elevation (31, 32). This post-treatment shift in NER underscores the precision of biologic action and its capacity to modulate inflammation without inducing broad immunosuppression. Importantly, eosinophils play roles beyond inflammation, including maintaining mucosal barrier function, yet current evidence indicates no significant hematological safety concerns with standard treatment durations (31–33).

In this study, we examined the evolving utility of blood biomarkers in severe asthma following biologic therapy. While biologics led to a marked decline in blood eosinophil count (BEC), this reduction did not correlate with improved asthma control or reduced exacerbations. Instead, higher neutrophil and monocyte counts, along with elevated NER, emerged as stronger indicators of persistent disease activity and exacerbation risk. Remarkably, logistic regression identified both NER and monocyte count as independent predictors of exacerbations, and ROC curve analysis supported their modest but significant predictive accuracy. These findings suggest a shift in biomarker relevance after biologic initiation, highlighting NER and monocyte count as alternative markers for ongoing inflammation and potential tools for risk stratification. Importantly, while these biomarkers are associated with exacerbation risk, they should not be used as standalone predictors; rather, they may serve as adjunctive tools, complementing patient history, clinical assessment, and other biomarkers to guide individualized management.

Moreover, despite effective eosinophil suppression, many patients continue to experience poor asthma control and exacerbations, suggesting that BEC alone may not capture the full spectrum of airway inflammation. BEC primarily reflects the pharmacodynamic effect of eosinophil-targeted therapies but does not account for non-eosinophilic or mixed inflammatory patterns (18). Elevated neutrophil and monocyte counts have been linked to worse asthma control and are increasingly recognized as hallmarks of neutrophilic or steroid-resistant asthma phenotypes (34). Integrating NER and neutrophil count into clinical assessment allows for a more comprehensive evaluation of residual inflammation, helping to identify patients who may require additional or alternative therapeutic strategies (18, 34). In this study, the selection of cutoff points for NER and monocyte count was based on balancing sensitivity and specificity to inform different clinical objectives. Lower thresholds (NER >3.97, monocytes >435) were chosen to maximize sensitivity, making them useful for screening or ruling out patients at risk of exacerbation, while higher thresholds (NER >50.65, monocytes >755) were selected to maximize specificity, allowing identification of patients at higher risk and confirming exacerbation likelihood. Although the higher cutoffs appear extreme, they illustrate the principle that the optimal threshold depends on the intended clinical application. This approach highlights how these biomarkers can be applied flexibly in practice, either to rule out exacerbation risk in low-risk patients or to confirm risk in those with more severe disease, despite the overall modest predictive accuracy indicated by the ROC analysis.

Moreover, after biologic initiation, traditional biomarkers like BEC lose their prognostic value for exacerbations due to eosinophil depletion (35). Instead, higher neutrophil and monocyte counts, as well as elevated NER, become more reliable predictors of continued exacerbation risk (35–37). Importantly, the observed rise in NER following biologic initiation may partly reflect a relative shift driven by eosinophil depletion, rather than solely an absolute increase in neutrophil-driven inflammation. Nonetheless, this change highlights the persistence of non-eosinophilic pathways, possibly linked to innate immune dysregulation or steroid-resistant mechanisms not targeted by current biologics (36, 37)

In this background, our findings support the potential clinical integration of NER and monocyte count into follow-up strategies for patients on biologics. Elevated levels of these markers may indicate residual inflammatory activity and help stratify patients into high- and low-risk groups. For high-risk individuals, more frequent monitoring of lung function and inflammatory biomarkers may be warranted. In cases where NER or monocyte count remains elevated, treatment modifications, such as targeting neutrophilic inflammation, reassessing corticosteroid responsiveness, or evaluating comorbidities, should be considered. These markers could be part of a broader biomarker panel, supporting personalized asthma care and more efficient resource allocation. While high sensitivity is essential for routine screening, high specificity is critical when making treatment changes to minimize unnecessary interventions. Mechanistically, elevated monocytes suggest innate immune activation that sustains steroid-resistant inflammation, reinforcing the need to address non-eosinophilic drivers in refractory asthma (38). However, these associations should be considered hypothesis-generating, and larger multi-center studies are required to validate their predictive value and confirm their applicability in broader clinical practice.

### Strength and limitation

This real-world study evaluated blood-based biomarkers in severe asthma patients receiving biologic therapy, offering insights into the effects of treatment on both eosinophilic and non-eosinophilic inflammation through the analysis of neutrophils, monocytes, the neutrophil-to-eosinophil ratio (NER), and blood eosinophil count (BEC). The application of correlation testing, logistic regression, and ROC analysis strengthened the evidence for NER and monocyte count as predictors of exacerbation risk, supporting their potential utility in post-biologic monitoring. Nevertheless, several limitations should be acknowledged. The observational design restricts causal inference, and the modest sample size may limit generalizability. Additionally, the predictive thresholds for NER and monocyte count were derived from our cohort, and external validation is warranted to confirm their applicability to other populations. Reliance on peripheral blood rather than airway-derived samples, a relatively short follow-up period, and the absence of environmental exposure data further constrain interpretation. In addition, although subgroup analyses by biologic type were considered, the small numbers of patients treated with mepolizumab and benralizumab limited statistical power. Consequently, the findings primarily reflect outcomes among patients treated with omalizumab and dupilumab. Future studies with larger, more balanced cohorts and longer follow-up are warranted to validate these observations and explore biomarker dynamics across different biologics.

## Conclusion

This study highlighted the evolving landscape of inflammatory biomarkers in severe asthma, particularly following biologic therapy. While biologics effectively suppressed eosinophilic inflammation, traditional markers such as BEC no longer reflected disease control or exacerbation risk. In contrast, elevated neutrophil and monocyte counts, along with an increased NER, emerged as potential indicators of persistent disease activity and exacerbation vulnerability. These findings suggest that incorporating NER and monocyte count into routine monitoring may enhance personalized care by identifying patients with residual non-eosinophilic inflammation, guiding treatment optimization beyond eosinophil-targeted strategies. However, these associations should be regarded as hypothesis-generating, and validation in larger, multi-center cohorts is essential before these biomarkers can be reliably implemented in clinical practice.

## Data Availability

The original contributions presented in the study are included in the article/Supplementary Material, further inquiries can be directed to the corresponding author.
